# EGFR Inhibition Strongly Modulates the Tumour Immune Microenvironment in EGFR-Driven Non-Small-Cell Lung Cancer

**DOI:** 10.3390/cancers14163943

**Published:** 2022-08-16

**Authors:** Carolin Selenz, Anik Compes, Marieke Nill, Sven Borchmann, Margarete Odenthal, Alexandra Florin, Johannes Brägelmann, Reinhard Büttner, Lydia Meder, Roland T. Ullrich

**Affiliations:** 1 Department I of Internal Medicine, Center for Integrated Oncology Aachen Bonn Cologne Duesseldorf, Faculty of Medicine and University Hospital Cologne, University of Cologne, 50931 Cologne, Germany; 2 Center for Molecular Medicine Cologne, Faculty of Medicine and University Hospital Cologne, University of Cologne, 50931 Cologne, Germany; 3 Mildred Scheel School of Oncology Cologne, Faculty of Medicine and University Hospital Cologne, University of Cologne, 50931 Cologne, Germany; 4 Institute of Pathology, Faculty of Medicine and University Hospital Cologne, University of Cologne, 50931 Cologne, Germany; 5 Department of Translational Genomics, Faculty of Medicine and University Hospital Cologne, University of Cologne, 50931 Cologne, Germany

**Keywords:** NSCLC, EGFR, tyrosine kinase inhibitors, immune checkpoint blockade, anti-PD-1, erlotinib, tumour microenvironment, immune cell infiltrate, immune response

## Abstract

**Simple Summary:**

Lung cancer that is driven by mutations in the epidermal growth factor receptor (EGFR) is currently treated with tyrosine kinase inhibitors (TKIs). Although patients initially respond well to TKI treatment, drug resistance against EGFR-targeted therapy emerges. Attempts to combine immunotherapy with EGFR-targeted treatment to prolong response rates or prevent the development of resistances have been limited due to insufficient knowledge about the effects of targeted therapy on the tumour microenvironment (TME) in EGFR-driven tumours and tumour-infiltrating immune cells. The aims of this study were to improve our understanding on the impact of EGFR inhibition on the immune response in EGFR-driven lung cancer and, furthermore, to gain insights into the impact of combining targeted therapy with immunotherapy on the TME.

**Abstract:**

EGFR-driven non-small-cell lung cancer (NSCLC) patients are currently treated with TKIs targeting EGFR, such as erlotinib or osimertinib. Despite a promising initial response to TKI treatment, most patients gain resistance to oncogene-targeted therapy, and tumours progress. With the development of inhibitors against immune checkpoints, such as PD-1, that mediate an immunosuppressive microenvironment, immunotherapy approaches attempt to restore a proinflammatory immune response in tumours. However, this strategy has shown only limited benefits in EGFR-driven NSCLC. Approaches combining EGFR inhibition with immunotherapy to stimulate the immune response and overcome resistance to therapy have been limited due to insufficient understanding about the effect of EGFR-targeting treatment on the immune cells in the TME. Here, we investigate the impact of EGFR inhibition by erlotinib on the TME and its effect on the antitumour response of the immune cell infiltrate. For this purpose, we used a transgenic conditional mouse model to study the immunological profile in EGFR-driven NSCLC tumours. We found that EGFR inhibition mediated a higher infiltration of immune cells and increased local proliferation of T-cells in the tumours. Moreover, inhibiting EGFR signalling led to increased activation of immune cells in the TME. Most strikingly, combined simultaneous blockade of EGFR and anti-PD-1 (aPD-1) enhanced tumour treatment response in a transgenic mouse model of EGFR-driven NSCLC. Thus, our findings show that EGFR inhibition promotes an active and proinflammatory immune cell infiltrate in the TME while improving response to immune checkpoint inhibitors in EGFR-driven NSCLC.

## 1. Introduction

Lung cancer is responsible for the most cancer-related deaths worldwide, with NSCLC accounting for nearly 80% of all cases [1,2]. Different subclassifications of NSCLC are identified by specific genetic alterations present in tumours, such as oncogenic driver mutations in the *epidermal growth factor receptor* (*EGFR*) gene. Targeted therapies against driver gene mutations have been developed and successfully established in the clinic in the form of EGFR TKIs, including erlotinib. TKI therapy has replaced standard chemotherapy as first-line treatment in EGFR-driven NSCLC due to promising initial response rates and prolonged progression-free survival of patients [3]. However, patients successfully treated with TKIs often become resistant to therapy after 9–14 months, most commonly by acquiring secondary EGFR mutations and, therefore, diminishing TKI efficacy [4,5]. Thus, novel therapy approaches are urgently required.

Increasing evidence demonstrates that, in NSCLC, the TME is generally characterised by noninflamed tumours with poor immune cell infiltrate mediated by immunosuppressive signals. This is attributed to different factors, such increased levels of inhibitory checkpoints on immune cells and their ligands that suppress antitumour activity [6,7]. This limits immunological surveillance and allows the tumour to progress and evade an active immune response [8]. In recent years, immunotherapy as an alternative treatment strategy has demonstrated beneficial antitumour responses in patients by mobilising the immune system to actively combat tumour cells that have previously escaped an immune response. By blocking key receptors facilitating inhibitory signalling pathways of the immune system, such as programmed cell death-1 (PD-1) and programmed cell death-ligand 1 (PD-L1), immune checkpoint blockade (ICB) has demonstrated encouraging responses in NSCLC patients [9,10]. However, when examining the response of EGFR-driven NSCLC patients specifically, ICB shows only limited benefits, for which the exact underlying mechanism still needs to be elucidated [9,11,12]. 

Different efforts have been made to boost efficacy of ICB in EGFR-driven NSCLC by combining it with target therapy. While a phase I clinical trial with advanced EGFR-driven NSCLC patients indicated durable tumour response rates upon combining ICB with erlotinib [13], the benefit of using this combination therapy approach is still not conclusively proven. This is illustrated by contrasting findings from preclinical studies investigating EGFR-driven NSCLC in mice. While Sugiyama and colleagues did observe an improved response upon application of both ICB and TKI treatment [14], implying potential benefits for combining ICB and EGFR inhibition, another study did not yield the same improved results after a four week period of simultaneous ICB and erlotinib therapy [15]. It should be noted that the administration of the therapy regimes, as well as the models used to mimic EGFR-driven NSCLC in vivo, varied between each study. These aspects should be taken into consideration when evaluating and comparing previous findings on the effect of ICB and targeted therapy on EGFR-driven tumours and the TME. Similarly, findings about the effect of EGFR inhibition on the immune cell infiltrate in these tumours have also displayed variability and remain to be conclusively established, with one study observing a decrease in regulatory T-cell (Tregs) levels after erlotinib therapy [14], whereas another did not notice any difference in tumour-infiltrating Tregs [15]. These contrasting findings not only illustrate the increasing need to further explore combinatorial approaches with ICB and TKI treatment, and their effect on EGFR-driven tumours, but also demonstrate how much is yet to be determined about the impact of targeted therapy alone on the TME and specifically on the immune cell infiltrate [16]. Therefore, to advance the understanding of the effects of TKI treatment on the immune cell infiltrate and improve upon existing therapy strategies, we investigate how EGFR inhibition modulates the TME in EGFR-driven NSCLC.

## 2. Materials and Methods

### 2.1. In Vivo Experiments

Experiments were performed in accordance with FELASA recommendations. The protocol was approved by the local ethics committee. Mice were housed and all experiments were performed in a sterile environment. Mice were fed, given water, and monitored daily for health, and cages were changed weekly.

### 2.2. Autochthonous EGFR^L858R^ NSCLC Model

We used a previously described EGFR-driven NSCLC mouse model [17,18]. *CCSP-rtTA; TetO-EGFR^L858R^* mice aged 8–16 weeks were fed ad libitum with doxycycline-containing feed (1000 ppm; ssniff Spezialdiäten GmbH) for the duration of the experiments. Four weeks after starting doxycycline feed, mice were scanned by μCT to confirm tumour formation. Tumour progression was monitored by weekly μCT scans using a LaTheta LCT-100 small animal μCT (Hitachi Aloka Instruments, Tokyo, Japan). CT images of the whole lung were taken at 0.3 mm intervals and analysed using Onis 2.5 Free Edition software (Digital Core Co., Ltd., Tokyo, Japan). Tumour progress and response were assessed by mouse-adapted RECIST criteria v1.1, as previously published [19]. The average of two tumour lesions per mouse was calculated and used to analyse tumour size fold change after therapy start. Tumour and spleen tissues were harvested at end of experiment and flash-frozen for subsequent RNA isolation, as well as further treated to obtain flow cytometry data. Overall survival of mice was assessed using Kaplan–Meier analysis.

### 2.3. Therapy Administration

Erlotinib (LC Laboratories, Woburn, MA, USA) was solved in 6% Captisol solution and orally administered at a concentration of 50 mg/kg body weight (BW) twice per week. Anti-mouse PD-1 antibody (clone RMP1-14, BioXCell, Lebanon, NH, USA) was administered at 10 mg/kg BW intraperitoneal twice per week [20,21]. Vehicle mice were treated twice per week with 6% Captisol given orally in combination with intraperitoneal administration of the appropriate murine aPD-1 IgG control (Isotype control rat IgG2a, κ; BioXCell, Lebanon, NH, USA). Mice were randomly assigned to the different therapy groups before start of treatment.

### 2.4. RNA Sequencing

RNA was extracted from flash-frozen tumour tissue by homogenisation using sterile 1.5 mL tissue homogenizers and subsequent isolation using Qiagen RNeasy Mini Kit, according to manufacturer’s protocol (Qiagen, Germantown, MD, USA). Corresponding to the manufacturer’s requirements, 20–30 mg of tissue was used from each sample. Libraries of 3’mRNA were obtained from total RNA using the Lexogen QuantSeq kit according to standard protocol. After validation and quantification (2200 TapeStation, Agilent Technologies, Santa Clara, CA, USA and Qubit System, Invitrogen, Carlsbad, California, CA, USA respectively), pools of cDNA libraries were generated. Pools were quantified using the KAPA Library Quantification kit (Peqlab, Radnor, PA, USA) and the 7900HT Sequence Detection System (Applied Biosystems, Foster City, PA, USA) and lastly sequenced on an Illumina HiSeq4000 or NovaSeq6000 sequencer using a 1 × 50 base pair protocol.

### 2.5. RNA Analysis

FASTQ files of 3′ UTR RNA-sequencing were checked for quality using FastQC (version 0.11.4), and reads were mapped to the mouse reference genome GRCm38 (p6) using the STAR aligner (version 2.7.0). Prior to downstream analysis, expression was quantified with RSEM (version 1.3.1). Analyses were run on the computing cluster of the Regional Computing Centre of the University of Cologne (RRZK). Gene set enrichment analysis (GSEA) was performed using GSEA software (version 4.0.2, Broad Institute, Cambridge, MA, USA). The *z*-scores of counts per million (CPM) were used as input, and all erlotinib-treated samples (namely, erlotinib and aPD-1 + erlotinib samples) were analysed against all other samples (vehicle and aPD-1 samples). To focus on gene sets relevant for certain aspects of immune function in the context of cancer, we used curated gene sets based on the ncounter Mouse PanCancer Immune Profiling Panel (Nanostring, Seattle, WA, USA), as previously established in our lab (Borchmann et al., under revision). Analyses were run with 1000 permutations, excluding gene sets smaller than five genes. Otherwise, standard settings were applied. Volcano plots of protein-coding transcripts were obtained after running multiple-comparison *t*-tests on sample *z*-scores from each treatment group against vehicle group samples.

### 2.6. Inference of TME Based on Differential Gene Expression

To interpret the composition of the tumour immune infiltrate on the basis of gene expression, we created a curated list of immune cell subtype-specific transcripts (Table S1). We started with a list of immune cell subtype enriched genes as defined in the Nanostring Vantage 3D RNA: Protein Immune Cell Profiling Assay (Nanostring, Seattle, WA, USA). This list was simplified by omitting transcripts enriched in multiple immune cell subtypes, only keeping transcripts unique to a specific immune cell subtype (Table S1). For each experiment, *z*-scores of CPM were calculated for all transcripts. The *z*-scores for all transcripts unique to a specific immune cell subtype from each sample were compared between groups to identify differences in the cellular composition of the tumour immune infiltrate.

### 2.7. Flow Cytometry

Single-cell suspensions of tumour and spleen tissues were generated via mechanical dissociation using 40 μm filters and taking up the cells in PBS. After pelleting cells, they were resuspended in 1 mL of ACK lysis buffer and incubated at room temperature for 5 min. Cells were washed once with PBS before proceeding with extracellular antibody staining and applying the viability dye Zombie UV (Biolegend, San Diego, CA, USA) for 30 min at 4 °C in FACS buffer (PBS containing 2% FBS and 1 mM EDTA). After incubation with antibodies, cells were washed with FACS buffer and fixed using 1% formaldehyde in FACS buffer for 15 min at room temperature. Permeabilisation of cells was performed using 0.1% Triton-X100-containing FACS buffer while incubating for 20 min at 4 °C. Subsequently, cells were stained with intracellular antibodies diluted in FACS buffer and incubated for 30 min at 4 °C. Before final analysis, cells were washed and resuspended in FACS buffer. The following extracellular antibodies (Biolegend, San Diego, CA, USA) were used for the analysis of cells: anti-CD45-PerCP-Cy5.5 (clone 30-F11), anti-CD3-PE-Cy7 (clone 145-2C11), anti-NK1.1-AF700 (clone PK136), anti-γδTCR-APC-Fire750 (clone GL3), anti-CD8a-BV421 (clone 53–6.7), anti-CD279 (PD-1)-BV510 (clone 29F.1A12), and anti-CD4-BV785 (clone GK1.5). The following intracellular antibodies (Biolegend, San Diego, CA, USA) were used: anti-IFNγ-PE-Dazzle594 (clone XMG1.2) and anti-Ki67-BV605 (clone 16A8). Flow cytometry of stained cells was performed on the Cytoflex LX Flow Cytometer (Beckmann Coulter, Krefeld, Germany). Results were analysed using Kaluza Software (version 2.1, Beckmann Coulter). Cells were initially gated to include alive singlet cells and then further selected using the following gating strategies. CD4^+^ T-cells were defined as CD45^+^CD3^+^CD4^+^ cells, while CD8^+^ T-cells were identified as CD45^+^CD3^+^CD8^+^ cells. NK T-cells were defined as CD45^+^CD3^+^NK1.1^+^ cells, and γδT-cells were defined as CD45^+^CD3^+^TCRγ/δ^+^ cells. IFNγ and Ki67 expression was assessed and reported as mean fluorescent intensity.

### 2.8. Cytokine Analysis

Serum samples were separated by centrifugation and stored at −80 °C until use. Levels of 32 murine biomarkers were quantified using a Mouse Cytokine/Chemokine 31-Plex Discovery Assay^®^ Array (Milipore, Darmstadt, Germany), according to the manufacturer’s protocol, and measured using the Luminex^TM^ 100 system (Luminex, Austin, TX, USA) by Eve Technologies Corporation (Calgary, AB, Canada). The biomarkers measured include Eotaxin, G-CSF, GM-CSF, IFNγ, IL-1α, IL-1β, IL-2, IL-3, IL-4, IL-5, IL-6, IL-7, IL-9, IL-10, IL-12p40, IL-12p70, IL-13, IL-15, IL-17A, IP-10, KC, LIF, LIX, MCP-1, M-CSF, MIG, MIP-1α, MIP-1β, MIP-2, RANTES, TNFα, and VEGF-A. All samples were measured in duplicate. Rarely, a detected marker was below the limit of quantification. In these instances, the value was set to half of the minimum quantification level. To generate a heatmap with Morpheus (Broad Institute, Cambridge, MA, USA), *z*-scores of the log_10_ of raw biomarker levels were calculated for each biomarker, and the heatmap was created using hierarchical clustering with the metric of 1 − Pearson correlation.

### 2.9. Immunohistochemistry

Lung tissue was harvested and fixed in 4% formaldehyde for 24 h, transferred to PBS, and embedded in paraffin (FFPE) using established routine protocols of the Pathology Department, University Hospital Cologne. Three micrometre lung sections were deparaffinised, and immunohistochemistry was performed on the LabVision Autostainer 480S (Thermo Fisher Scientific, Waltham, MA, USA). Staining was performed using haematoxylin and eosin (H&E), as well as primary antibodies against EGFR^L858R^ (Cell Signaling Technologies, 3197, Leiden, The Netherlands), CD3 (Thermo Fisher, RM-9107-S, Waltham, MA, USA), CD4 (Abcam, EPR19514, Cambidge, UK), CD8 (Abcam polyclonal, ab203035, Cambidge, UK), and CD45R (BD Biosciences, 550286, Franklin Lakes, NJ, USA). Subsequently, primary antibodies were detected using secondary Histofine Simple Stain (SHSS) detection kits (Medac, Wedel, Germany). Slides were scanned on the Leica SCN400 Slidescanner (Leica Biosystems, Deer Park, IL, USA).

### 2.10. Statistics

Statistical analyses and data graphs were carried out using GraphPad Prism (version 8.4.3), unless stated otherwise. Statistical tests were performed as described in figure legends.

## 3. Results

### 3.1. Inhibition of EGFR Mediates Higher Immune Cell Infiltration in the TME of EGFR-Driven Tumours

**Figure 1:**
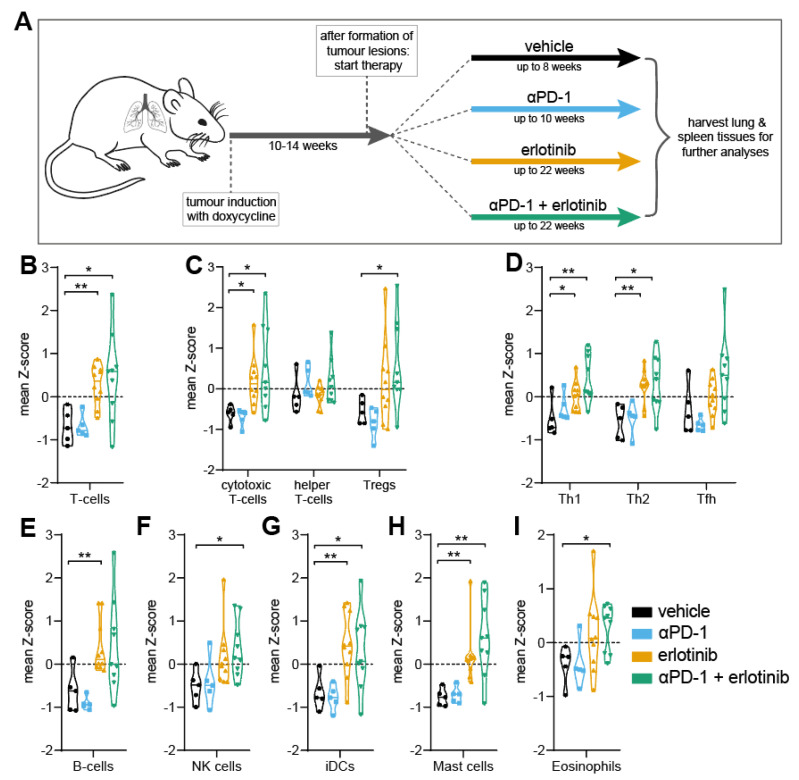
Inhibition of EGFR mediates higher immune cell infiltration in the TME of EGFR-driven tumours. (**A**) Experimental setup of tumour induction and treatment strategies using EGFR^L858R^-NSCLC mice on a continuous doxycycline diet. Mice were divided into different therapy cohorts: vehicle, αPD−1, erlotinib, or αPD−1 + erlotinib. After up to 22 weeks under therapy, lung tumours and spleens were harvested for further analysis, including RNA isolation or flow cytometry. (**B**–**D**) Immune cell deconvolution illustrating mean gene expression *z*-scores of T-cell-specific transcripts (*n* = 5–12 mice per group). (**E**–**I**) Immune cell deconvolution illustrating mean gene expression *z*-scores of immune cell-specific transcripts (*n* = 5–12 mice per group). (**B**–**I**) Data are shown as violin plots; the statistical test used was Student’s *t*-test (statistically significant changes are indicated as follows: * *p* < 0.05; ** *p* < 0.01).

To investigate the changes in the TME occurring after EGFR inhibition via TKI therapy, we treated autochthonous EGFR^L858R^-driven NSCLC mice with vehicle, aPD-1, erlotinib, or aPD-1 + erlotinib to assess and compare the effects of both immunotherapy and targeted therapy approaches (Figure 1A). To distinguish the effect of EGFR inhibition on the TME, we applied immune cell deconvolution via 3′ mRNA-sequencing. Comparing lesions treated with erlotinib to vehicle- or aPD-1-treated tumour samples, we observed a significant increase in intratumoural T-cell levels in the erlotinib and aPD-1 + erlotinib group (Figure 1B). Upon investigating different subtypes of T-cells, we specifically detected higher infiltration of not only cytotoxic CD8^+^ T-cells, but also of Th1, Th2, and Tfh cells upon EGFR inhibition (Figure 1C,D). Interestingly, we also observed increased levels regulatory T-cells (Tregs) in the TME of tumours treated with erlotinib and aPD-1 + erlotinib (Figure 1C). In addition to higher levels of intratumoural T-cells, we further detected overall increased infiltration of natural killer (NK) cells, mast cells, and eosinophils upon EGFR inhibition, as well as higher levels of intratumoural B cells and dendritic cells (DCs), thus increasing antigen capabilities of the TME (Figure 1E–I). Greater infiltration of T-cells in general, as well as CD8^+^ T-cells and B cells, in the TME upon EGFR inhibition was further observed in immunohistochemistry staining of tumour sections from samples treated with either erlotinib or aPD-1 + erlotinib (Figure S1). Taken together, these data demonstrate an overall greater infiltration of immune cells into the TME facilitated by EGFR inhibition.

### 3.2. EGFR Inhibition Enhances Proliferation and Activation of T-Cells in TME

**Figure 2:**
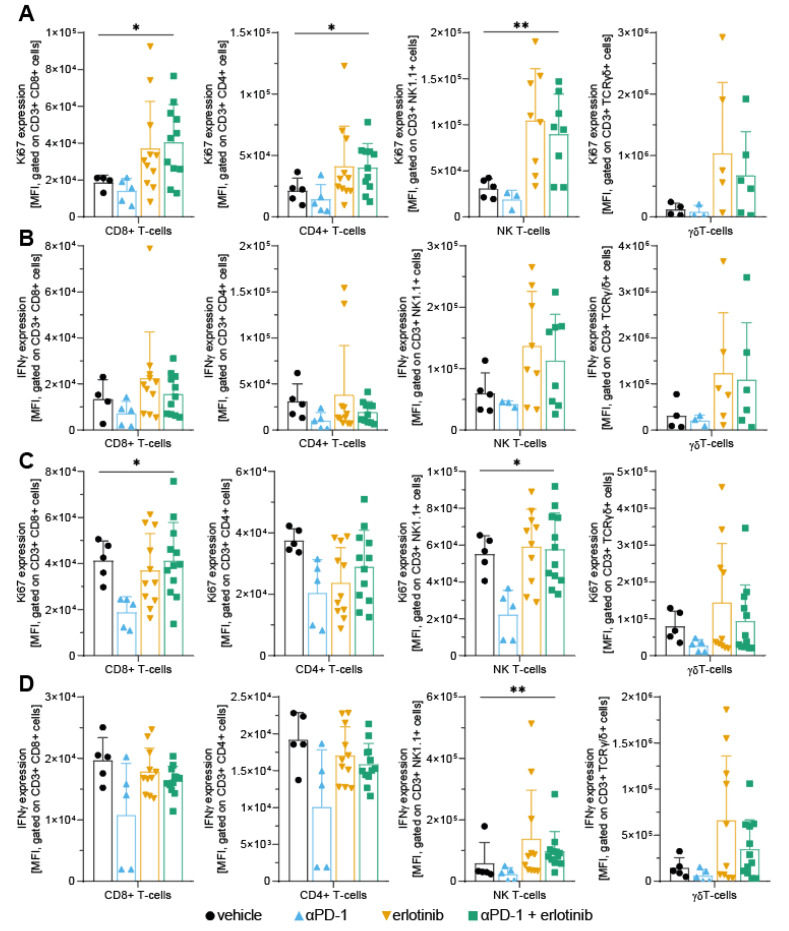
EGFR inhibition enhances proliferation and activation of T-cells in TME. (**A**,**B**) Mean fluorescence intensity data of (**A**) intracellular Ki67 and (**B**) intracellular IFNγ expression, illustrating proliferation and activation status, respectively. Data shown for cytotoxic CD8^+^ T-cells, helper CD4^+^ T-cells, NK T-cells, and γδT-cells from lung tumour tissue. (**C**,**D**) Mean fluorescence intensity data of (**C**) intracellular Ki67 and (**D**) intracellular IFNγ expression, illustrating proliferation and activation status, respectively. Data shown for cytotoxic CD8^+^ T-cells, helper CD4^+^ T-cells, NK T-cells, and γδT-cells from spleen tissue (*n* = 5–12 mice per group). (**A**–**D**) Data are shown as the mean with SD; the statistical test used was the Kruskal–Wallis test to compare all therapy groups (statistically significant changes are indicated across all groups as follows: * *p* < 0.05; ** *p* < 0.01).

After detecting increased levels of immune cells in the TME, we further explored the functional dynamics of the immune cell infiltrate to ascertain the effect of EGFR inhibition on the inflammatory status of the TME. To assess T-cell activity and proliferative capacity, single-cell suspensions of lung tumour tissue were analysed by flow cytometry. Notably, we observed that Ki67 expression levels of all T-cell subtypes were increased in tumours that were treated with erlotinib (Figure 2A). A similar trend was detected for the combination group treated with ICB and erlotinib. In contrast, we observed a slight decrease in Ki67 expression for ICB-treated mice compared to the vehicle group, indicating lower proliferation of T-cells upon aPD-1 monotherapy (Figure 2A). Regarding IFNγ expression, we noticed similar trends of increasing levels in tumour tissue upon EGFR inhibition in the erlotinib-treated mice. Again, this trend was not only observed in the erlotinib monotherapy group, but also when combined with aPD-1, indicating increased activation in T-cells, especially in the NK T-cell and γδT-cell populations, of the TME. Similar to Ki67 expression, IFNγ levels in tumours treated with ICB exhibited a slight decrease compared to vehicle tumours (Figure 2B). Concerning PD-1 expression, no changes in PD-1 levels were observed on the different T-cell subtypes, except for increased levels of PD-1 after ICB in CD8^+^ T-cells (Figure S2A). To ascertain whether the observed shifts in Ki67 and IFNγ expression in CD4^+^, CD8^+^, NK, and γδT-cells were locally specific to the TME, flow cytometry was performed using single-cell suspensions of spleen tissue from the treated mice. In contrast to our findings from the tumour immune infiltrate, we found that Ki67 levels were not altered upon EGFR inhibition compared to vehicle control in spleen tissues (Figure 2C). Moreover, we did not detect consistent changes in IFNγ expression in CD4^+^ and CD8^+^ T-cells after erlotinib treatment, as well as only a slight increase of IFNγ levels in NK and γδT-cells (Figure 2D). This suggests that increased proliferation and activation of T-cells in response to EGFR inhibition are specific to the TME. Notably, reduced expression of both Ki67 and IFNγ in aPD-1-treated mice compared to the vehicle group was more pronounced in the spleen than in tumour tissue (Figure 2C,D). These data suggest that ICB therapy alone, targeting the PD-1 signalling axis, conveys an immunosuppressive phenotype in T-cells, affecting T-cells not only in the TME, but also in other tissues. This was further confirmed after analyses of circulating cytokine signatures, which illustrated more pronounced changes in cytokine levels upon ICB treatment compared to vehicle control (Figure S3A–C). In contrast, circulating cytokine levels from mice treated with either erlotinib or aPD-1 + erlotinib did not indicate notable changes (Figure S3A–C). Together, these data reveal that EGFR inhibition appears to modulate T-cells specifically in the TME towards an enhanced proliferative and inflammatory phenotype, thus facilitating an improved T-cell response against the tumours.

### 3.3. Inhibition of EGFR Increases Active Phenotype of Immune Cell Infiltrate in EGFR-Driven Tumours

**Figure 3:**
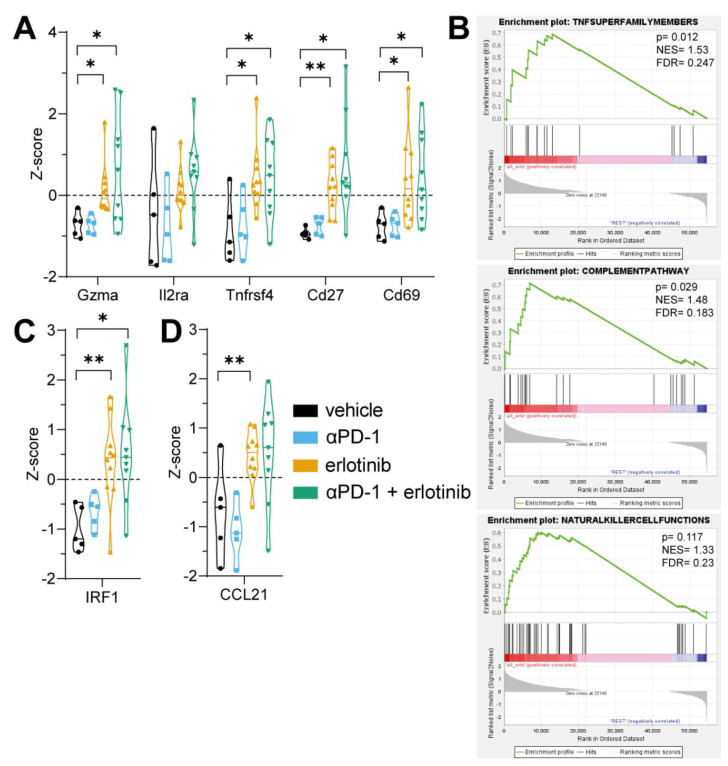
Inhibition of EGFR increases active phenotype of immune cell infiltrate in EGFR-driven tumours. (**A**) Mean gene expression *z*-scores of immune cell activation markers (*n* = 5–10 mice per group). (**B**) Gene set enrichment analysis for different gene sets from any erlotinib-treated mice (namely, erlotinib and aPD−1 + erlotinib groups) against the others (vehicle and aPD−1 groups). (**C**,**D**) Mean gene expression *z*-score of transcription factor mediating tumour-suppressive functions and intratumoural chemokines, respectively (*n* = 5–12 mice per group). (**A**,**C**,**D**) Data are shown as violin plots; the statistical test used was Student’s *t*-test (statistically significant changes are indicated as follows: * *p* < 0.05; ** *p* < 0.01).

Following our results of an increased inflammatory T-cell phenotype in the TME, we were prompted to examine the general activation status of the TME using 3′ mRNA-sequencing data. To determine, whether similar trends could be observed for the overall immune response in the TME, *z*-scores of transcripts increasingly expressed upon immune cell activation were examined. These transcripts included Gzma, Il2ra, Tnfrsf4, encoding granzyme A, IL-2, and OX40, as well as CD29 and CD69 (Figure 3A). These markers are not only known to be upregulated in active T-cells, but are also associated with proliferation and activation of other immune cell types, such as B cells and NK cells [22,23,24,25,26]. Interestingly, increased levels of activation-specific gene expression were observed in both erlotinib monotherapy and aPD-1 + erlotinib groups (Figure 3A), suggesting that EGFR inhibition mediates a shift towards a more inflammatory immune cell infiltrate. To further investigate which signatures of immune response are promoted by EGFR inhibition, gene set enrichment analysis (GSEA) was performed comparing all tumours undergoing EGFR inhibition (namely, erlotinib monotherapy and aPD-1 + erlotinib) against vehicle and aPD-1 tumours. After GSEA analysis, significant enrichment was detected in signatures promoting an active immune response (Figure 3B), including signatures of the TNF superfamily, which is known to promote a proinflammatory immune response and mediates signalling responsible for proliferation, differentiation, and effector functions of immune cells (Figure 3B) [27,28]. Moreover, the complement pathway has been implicated enhancing T-cell function and proliferation [29,30], and NK cells play a crucial role in antitumour response [31,32], illustrating the importance of enrichment in NK cell functions (Figure 3B). These observations further strengthen our findings that EGFR inhibition not only affects T-cell activity, but also stimulates an increased response of the immune cell infiltrate overall. To investigate potential mechanisms of how the modulation of oncogenic EGFR signalling in tumour cells facilitates changes in the immune response, we next examined the expression of the transcription factor, IRF1, a known tumour suppressor gene. Previously, IRF1 has been implicated not only in the regulation of CD274 (PD-L1) expression [33], but also in playing a role in suppressing tumour proliferation and stimulating an active immune response in tumours [34,35]. Moreover, IRF1 has been shown to be negatively regulated by oncogenic EGFR signalling in NSCLC [14]. In line with previous studies, IRF1 expression was increased in tumours, displaying a proinflammatory immune phenotype upon EGFR inhibition (Figure 3C). Increases in PD-L1 or PD-L2 levels were not observed, presumably due to the high spread of transcript expression in the vehicle group (Figure S2B). In contrast, expression of CCL21 was also elevated upon blocking oncogenic EGFR signalling (Figure 3D). CCL21 is a chemotactic cytokine known to recruit T-cells to the TME, thus promoting increased immune activity [36,37].

**Figure 4:**
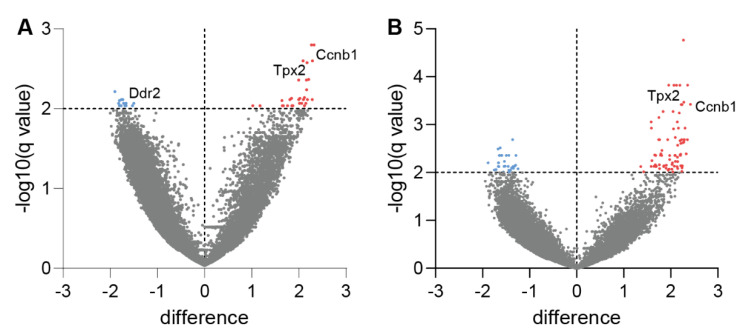
EGFR inhibition leads to increase in transcripts associated with higher immune cell infiltration. (**A**) Volcano plot showing transcripts detected at significantly altered levels in lung tumour tissue from erlotinib-treated mice compared to vehicle control group. (**B**) Volcano plot showing transcripts detected at significantly altered levels in lung tumour tissue from aPD−1 + erlotinib-treated mice compared to vehicle control group. (**A**,**B**) Blue points illustrate significantly downregulated transcripts, while red points indicate significantly upregulated transcripts.

Furthermore, analysis of the most differentially expressed transcripts by multiple-comparison *t*-tests of sample *z*-scores revealed multiple significantly upregulated transcripts in both erlotinib (Figure 4A) and aPD-1+erlotinib groups (Figure 4B) compared to vehicle. These include Ccnb1 and Tpx2, which have been previously associated with higher immune cell infiltration [38,39] and antitumour activity of CD8^+^ T-cells [40]. Interestingly, expression of Ddr2, a collagen receptor playing a key role in cell interaction, was significantly downregulated in the erlotinib-treated samples compared to control. Depletion of Ddr2 has been previously associated with higher CD8^+^ T-cell infiltration, as well as increasing sensitivity towards ICB [41]. Taken together, these data illustrate that blocking oncogenic EGFR signalling in tumours increased the immune cell infiltration in the TME and stimulated a proinflammatory immune response against tumours.

### 3.4. Simultaneous EGFR Inhibition and ICB Indicate Slower Tumour Growth and Improved Antitumour Response over EGFR Inhibition Alone in EGFR-Driven NSCLC Model

**Figure 5:**
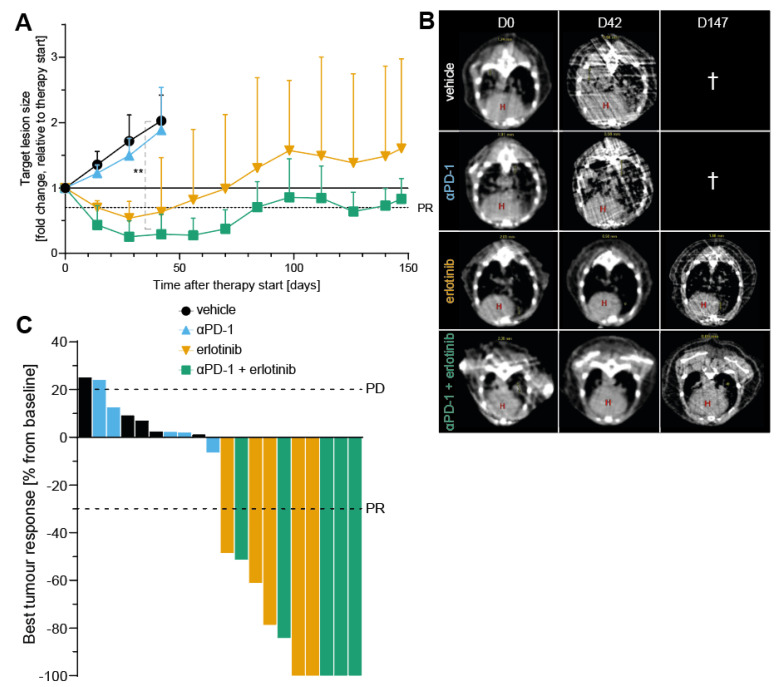
Simultaneous EGFR inhibition and ICB indicate slower tumour growth and improved antitumour response over EGFR inhibition alone in EGFR-driven NSCLC model. (**A**) Mean fold change of EGFR-driven target lesion growth over time in indicated treatment groups. Data are shown as the mean with SD; the statistical test used was Student’s *t*-test of individual groups at the endpoint of vehicle and aPD−1 groups (day 42; statistically significant changes are indicated as follows: ** *p* < 0.01). (**B**) Representative µCT images taken prior to therapy start (D0) and on days 42 (D42) and 147 (D147) after therapy start of EGFR^L858R^-driven mice; the red H indicates the heart; † indicates that no mice from treatment group reached the indicated time point. (**C**) Best response to therapy from beginning of treatment (baseline) for individual mice. (**A**,**C**) PR, partial response; PD, progressive disease.

On the basis of our observations that blocking EGFR signalling in oncogene-driven NSCLC alters the TME towards a proinflammatory status, thus promoting an enhanced immune response, we next examined tumour growth rates by analysing target lesion size to assess tumour response. Mice treated with aPD-1 alone showed no treatment response (Figure 5A,B), underlining the limited efficacy of ICB in EGFR-driven tumours [9,42]. In line with our previous results, EGFR inhibition improved antitumour response compared to vehicle (Figure 5A,B) and significantly increased overall survival of mice treated with erlotinib over both vehicle and aPD-1 groups (Figure S3D). Furthermore, combined treatment with aPD-1 + erlotinib also prolonged overall survival and improved tumour response to therapy compared to the vehicle and aPD-1 control groups (Figure 5A,B and Figure S3D). When analysing the best response rates, similar results for mice treated with erlotinib monotherapy or combining ICB with EGFR inhibition were observed (Figure 5C). Interestingly, we were able to detect a trend towards improvement of antitumour response in the combination group when considering target lesion data. This was indicated by not only a faster reduction in tumour size upon aPD-1 + erlotinib therapy compared to EGFR inhibition alone, but also by tumours remaining in a partial response (PR) state until the end of the experiments, in contrast to the erlotinib group (Figure 5A). To summarise, we observed compelling antitumour responses upon blocking oncogenic EGFR signalling with prolonged survival over vehicle tumours. When combining EGFR inhibition with ICB, we observed a trend to faster antitumour response and slower outgrowth of tumours after relapse, compared to EGFR inhibition alone.

## 4. Discussion

In this study, we aimed to improve our understanding of the limited efficacy of immune checkpoint inhibitors in EGFR-driven NSCLC patients and the role of oncogenic EGFR signalling for the immunosuppressive composition of the TME. This was approached by investigating the effect of EGFR inhibition through TKI erlotinib in an oncogene-dependent mouse model and examining different aspects of the corresponding immune response. It has been reported that oncogene-driven tumours are characterised by the establishment of a noninflamed TME, thus facilitating the evasion of an active immune response by tumours [8]. Major factors promoting an immunosuppressive TME include low infiltration of immune cells into the tumour, lack of proliferation to achieve an appropriate antitumour response, and/or reduced activation of tumour-infiltrated immune cells [43,44]. Previous studies have shown that, although immunotherapy approaches such as targeting immune checkpoint by ICB are beneficial in many cancer entities to promote a proinflammatory TME, EGFR-driven NSCLC tumours do not respond to this type of therapy [9,11,12]. Given the increasing interest in applying immunotherapy in combination with targeted therapies to try and circumvent this issue and increase efficacy of ICB in EGFR-driven NSCLC, different efforts have been made to investigate combination therapy regimes [13,14,15]. However, due to varying results on the effect of combining ICB with targeted therapy, no final conclusion on potential benefits of this therapy regime can be drawn to date. To appropriately assess the implication of combining immunotherapy with targeted therapy on the TME, it is also critical to consider how targeted therapy alone impacts not only tumour cells, but also other components of the TME. This question has gained more attention in recent years, but has not yet yielded a comprehensive answer, in part also due to varying results on the subject, such as differences between infiltrating immune cell populations [14,15,16]. This illustrates the need for further investigation in this field and validation of previous findings. In our study, we found that, in unresponsive tumours, where oncogenic EGFR signalling remained constitutively active, levels of immune cells in the TME were reduced compared to tumours that exhibited an active tumour response to therapy (Figure 1B–I), aligning with previous evidence that untreated EGFR-driven NSCLC mediates an immunosuppressive TME. In turn, we observed that, upon EGFR inhibition, immune cell infiltration was elevated in the TME (Figure 1B–I), confirming previous findings after erlotinib treatment in NSCLC mouse models [15,45]. Moreover, EGFR inhibition in tumour cells induced T-cell proliferation and activation, thus promoting an enhanced immune response in tumours treated with the TKI erlotinib (Figure 2A). This further confirms observations that erlotinib increases cytokine-producing T-cells in the TME [15]. As previously mentioned, aPD-1 therapy does not induce beneficial responses in patients with EGFR-driven tumours [9,11,12]. This corresponds with our findings, which did not indicate improvements in tumour response upon ICB treatment compared to vehicle control (Figure 5A–C). An explanation for these results could be the downregulation of PD-L1 on tumour cells, mediated indirectly by oncogenic EGFR signalling via IRF1. In previous studies, transcription factor IRF1 was shown to be negatively impacted by EGFR signalling [14,35]. This corresponds to our own data that illustrate an increase in IRF1 expression levels upon EGFR inhibition (Figure 3C). Additionally, IRF1 has also been implicated in the regulation of CD8^+^ T-cell infiltration in tumours by mediating the expression of the chemokine CXCL10 [14]. This could be one mechanism underlying how EGFR inhibition increases CD8^+^ T-cell infiltration in EGFR-driven tumours that we observed (Figure 1B and Figure S1). Another chemokine that has been linked to EGFR signalling is CCL21, previously shown to be involved in the recruitment and infiltration of tumour-specific T-cells into the TME [37]. CCL21 has been previously shown to be downregulated in EGFR-driven tumours and contribute to an immunosuppressive TME [14], which can be confirmed by our data (Figure 3D). Moreover, in hepatic cell cancer (HCC), expression of *Ccnb1* and *Tpx2* was positively associated with higher immune cell infiltration in tumours, suggesting potential immune-regulatory roles for the proteins [38,39]. This is line with recent results indicating that Tpx2 overexpression increased antitumour activity of CD8^+^ T-cells in HCC in a CXCR5-dependent manner [40]. Lastly, treatment with ICB in combination with EGFR inhibition induced an antitumour immune response, when considering levels of immune cell infiltrate (Figure 1B–I) and immune cell activation in the TME (Figure 3A). Despite similar immune cell infiltrate signatures between ICB combined with EGFR inhibition and EGFR inhibition alone, growth data suggest an improved antitumour response in aPD-1 + erlotinib mice, resulting in a faster reduction in tumour size and slower tumour progression until the end of the experiments (Figure 5A). However, this observation remains to be confirmed in future studies. 

In this study, we examined the immune-modulating effect of targeted therapy in EGFR-driven NSCLC on the TME using an EGFR^L858R^-dependent mouse model. Moreover, we provided insights into the changes to the TME after long-term exposure to continuous targeted therapy using erlotinib, as well as after ongoing simultaneous long-term administration of targeted therapy and ICB in EGFR-driven NSCLC. We observed that EGFR inhibition induces a proinflammatory immune response with increased proliferation and activation of tumour-infiltrated T-cells. Thus, our results suggest that oncogenic EGFR signalling modulates the TME to evade the immune response by promoting an immunosuppressive microenvironment. Future studies should provide more insights into the underlying cellular mechanisms involved in this process.

## 5. Conclusions

In summary, our findings shed more light on the impact of targeted therapy on the TME, as well as improve our understanding of how the immune cell infiltrate is altered upon continuous long-term exposure to TKI treatment and simultaneous administration of TKIs and ICB in EGFR-driven tumours. These aspects are not only critical for improving current targeted therapy approaches, but also provide important and clinically relevant information for future investigations on combining immunotherapy with TKIs to further stimulate and improve the antitumour immune response in EGFR-driven NSCLC.

## Data Availability

The RNA sequencing data presented in this study are openly available in the NCBI Sequence Read Archive under the accession code PRJNA860558 (https://www.ncbi.nlm.nih.gov/sra/PRJNA860558, accessed on 20 July 2022).

## Supplementary Materials

The following supporting information can be downloaded at https://www.mdpi.com/article/10.3390/cancers14163943/s1: Figure S1. Infiltration of immune cells in EGFR^L585R^-driven tumours is elevated upon EGFR inhibition; Figure S2. EGFR inhibition does not increase PD-1 levels; Figure S3. EGFR inhibition does not increase circulating cytokines; Table S1. Immune cell-specific transcripts.
