# Supplementary material for: EGFR Inhibition Strongly Modulates the Tumour Immune Microenvironment in EGFR-Driven Non-Small-Cell Lung Cancer
Source: Cancers (Basel). 2022 Aug 16;14(16):3943. doi: 10.3390/cancers14163943 (PMC9406398; doi:10.3390/cancers14163943)
Supplement: Supplementary file 1 [file cancers-14-03943-s001.zip › cancers-1851890-supplementary.pdf]

cancers

MDPI

Supplementary materials

EGFR Inhibition Strongly Modulates the Tumour Immune Microenvironment in EGFR-Driven Non-Small-Cell Lung Cancer

Carolin Selenz [1,2], Anik Compes [1,2,3], Marieke Nill [1,2], Sven Borchmann [1], Margarete Odenthal [4], Alexandra Florin [4], Johannes Brägelmann [2,3,5], Reinhard Büttner [4], Lydia Meder [1,2,3*], and Roland T. Ullrich [1,2,*]

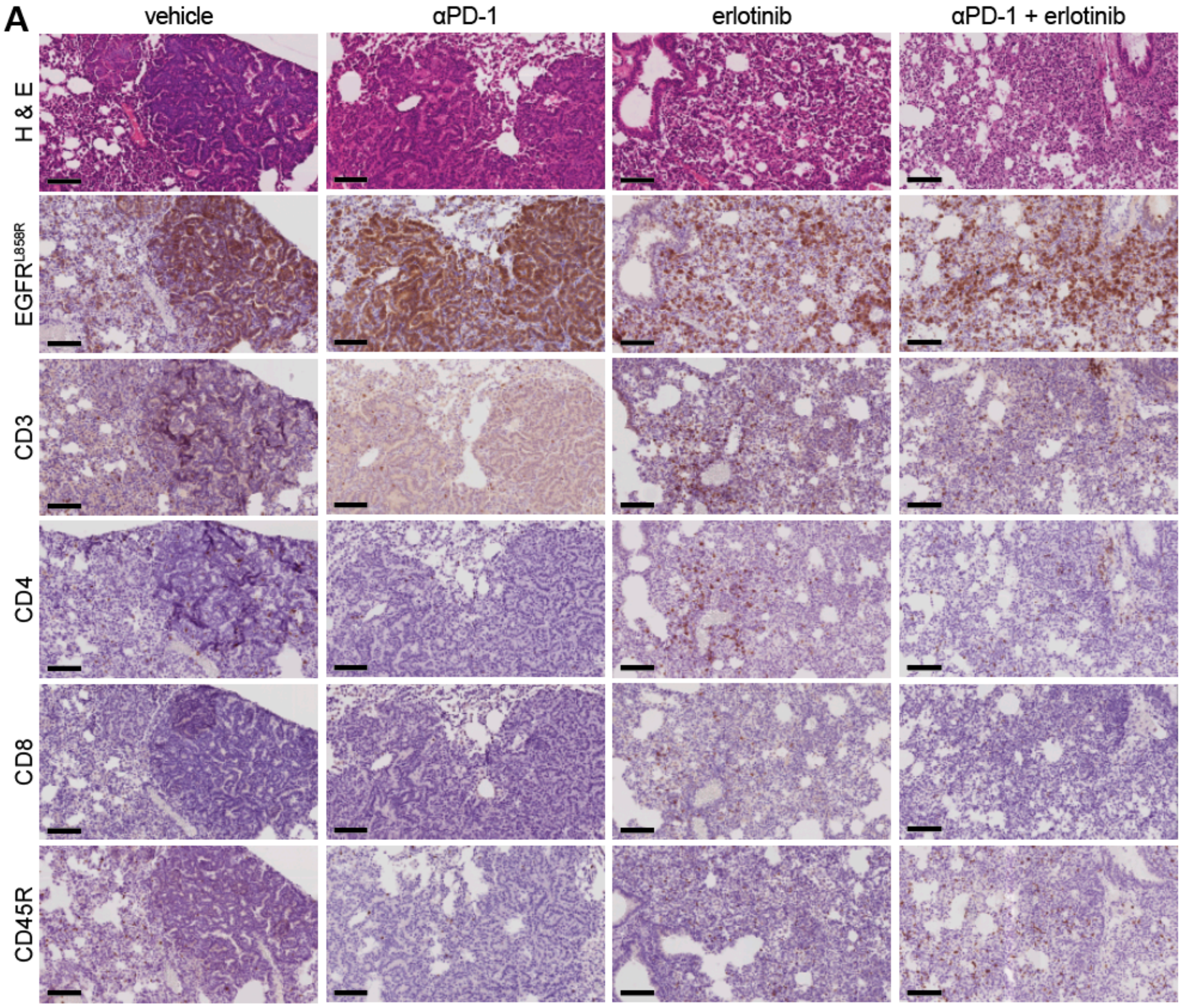

Figure S1. Infiltration of immune cells in EGFR^{L585R}-driven tumours is elevated upon EGFR inhibition. (**A**) Images from stained FFPE tumour material either after H&E or immunohistochemistry staining with the indicated antibodies, identifying EGFR$^{L8585R+}$ tumour cells, general T−cells, CD4$^+$ T−cells, CD8$^+$ T−cells and CD45$^+$ B−cells. One representative lesion from each therapy group is shown. Scale bars indicate 100 µm.

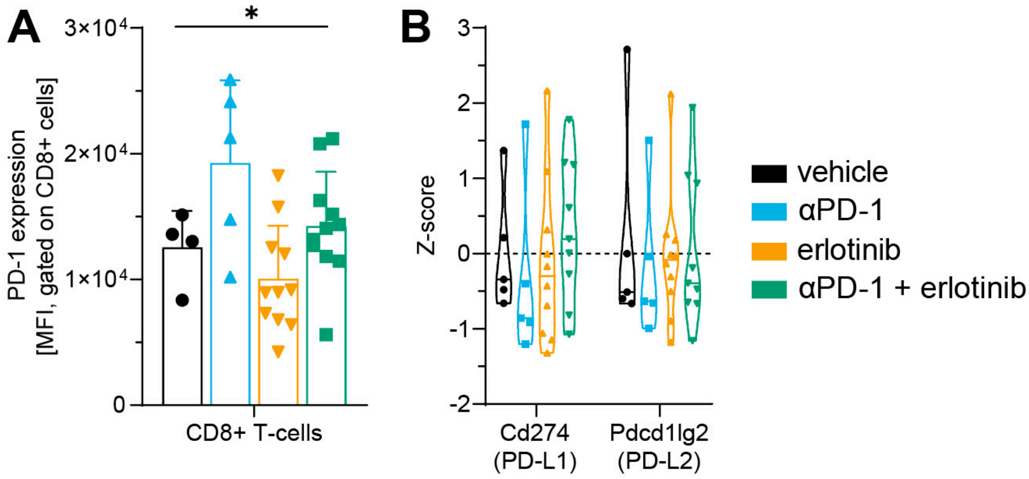

Figure S2. EGFR inhibition does not increase PD−1 levels. (**A**) Mean fluorescent intensity data of PD−1 expression of cytotoxic CD8^{+} T−cells. Data are shown as the mean with SD, statistical test used was the Kruskal−Wallis test to compare all therapy groups (statistically significant changes are indicated across all groups as follows: *, p < 0.05). (**B**) Mean gene expression *z*-score of Cd274 (PD−L1) and Pdcd1lg2 (PD−L2). Data are shown as violin plots.

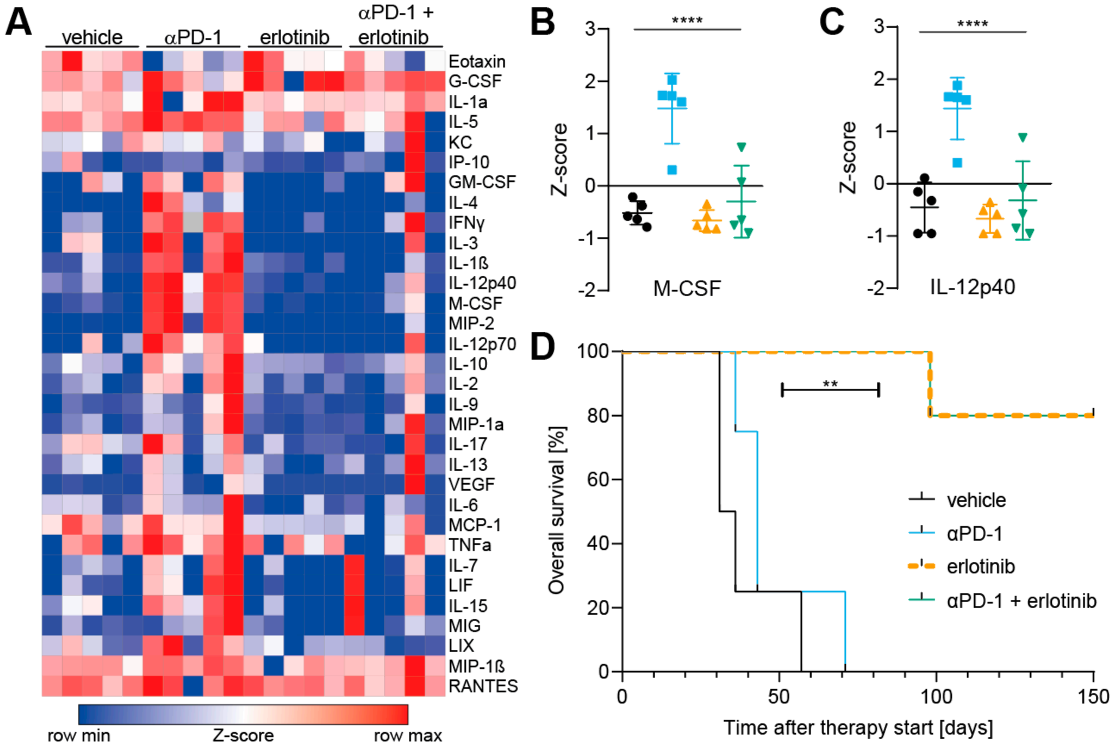

Figure S3. EGFR inhibition does not increase circulating cytokines. (**A**) Heatmap of *z*-scores of circulating cytokines quantified by multiplex Luminex analysis in sera of mice treated with vehicle, aPD−1, erlotinib or aPD−1 + erlotinib, as indicated. Colour gradient ranges from minimum (blue) to maximum (red) *z*-score value for each cytokine. (**B-C**) Circulating levels of cytokines (**B**) M−CSF and (**C**) IL12−p40 in sera of mice (**D**) Kaplan-Meier survival curve of mice bearing EGFR^{L858R}-driven lung tumours from indicated therapy groups (statistically significant changes are indicated as follows: **, p < 0.01; ****, p < 0.0001).

Table S1. Immune cell-specific transcripts.

Cell type	Genes							
mDC	CCL13	CCL17	CCL22	HSD11B1				
iDC	CD1A	CD1B	CD1E	F13A1	SYT17			
aDC	CCL1	EBI3	IDO1	LAMP3	OAS3			
pDC	IL3A							
Macrophages	APOE	CCL7	CD68	CHIT1	CXCL5	MARCO	MSR1	
Mast cells	CMA1	CTSG	KIT	MS4A2	PRG2	TPSAB1		
Neutrophils	CSF3R	FPR2	MME					
Eosinophils	CCR3	IL5RA	PTGDR2	SMPD3	THBS1			
B-cells	BLK	CD19	CR2	HLA-DOB	MS4A1	TNFRSF17		
T-cells	CD2	CD3E	CD3G	CD6				
Th1	CD38	CSF2	IFNG	IL12RB2	LTA	CTLA4	TXB21	STAT4
Th2	CXCR6	GATA3	IL26	LAIR2	PMCH	SMAD2	STAT6	
Th17	IL17A	IL17RA	RORC					
Tfh	CXCL13	MAF	PDCD1	BCL6				
Treg	FOXP3							
Cytotoxic CD8	CD8A	CD8B	FLT3LG	GZMM	PRF1			
gdTcells	CD160	FEZ1	TARP					
NK cells	BCL2	FUT5	NCR1	ZNF205				
CD56bright	FOXJ1	MPPED1	PLA2G6	RRAD				
CD56dim	GTF3C1	GZMB	IL21R					
